# Pollen foraging preferences in honey bees and the nutrient profiles of the pollen

**DOI:** 10.1038/s41598-024-65569-1

**Published:** 2024-07-01

**Authors:** Seiji C. Yokota, Corey Broeckling, Arathi H.S. (Seshadri)

**Affiliations:** 1 Invasive Species and Pollinator Health Research Unit, USDA-ARS/PWA/WRRC, Davis, CA 95616 USA; 2 Analytical Resources Core/Data Science Research Institute/Department of Agricultural Biology, Colorado State University, Fort Collins, CO 80523 USA; 3 Pollinator Health in Southern Crops Ecosystems Research Unit, USDA-ARS/SEA, Stoneville, MS 38776 USA

**Keywords:** *Apis mellifera*, Almonds, Mixed species plantings, Pollen metabolomics, Sunflower, Animal behaviour, Agroecology, Behavioural ecology

## Abstract

Honey bees are important insect pollinators that provide critical pollination services to fruit and nut crops in the US. They face challenges likely due to pressures associated with agricultural intensification related habitat loss. To better understand this, pollen preferences of foraging bees and the nutritional profile of pollen brought into hives by foraging bees in crop fields and nut orchards can provide valuable information. We trained bees to forage on bee-collected pollen from hives placed for pollination services in almond orchards, sunflower fields, or mixed species from inter-row plantings. Using bees trained to a certain kind of hive pollen, we applied a binary scoring system, to test preferences of these preconditioned foragers. We also performed metabolomic analyses of the hive pollen used for training and testing to elucidate their nutritional content. Irrespective of preconditioning, bees collected all the available choice pollen types, predominantly choosing hive-collected mixed species pollen (MSP), followed by almond orchard pollen. The hive-collected MSP was chemically diverse, richest in cholesterol, vitamins, and phytochemicals quercetin, kaempferol, coumarin, and quinine, but was not consistently high for essential amino acids and polyunsaturated fatty acids. Although diversity in chemical profiles may not directly relate to plant species diversity, our results suggest that foragers collect a variety of pollen types when available reiterating the importance of diverse floral resources.

## Introduction

As a major insect pollinator, the western honey bee, *Apis mellifera*, is essential for pollination services in fields of pollination-dependent fruits, nuts, oilseeds, and vegetables. Honey bee populations have been experiencing fluctuations over the past few decades with colony losses reported at over 50%^1–5^. *Varroa* mites, pathogens, poor nutrition due to habitat degradation, loss of genetic diversity, pesticide exposure and changing weather conditions have all been implicated as factors leading to this decline^3,6–9^. While honey bees are a near global species, the commercial pollination industry where thousands of honey bee colonies are brought each year to pollinate almonds in California, and the challenges these growers and beekeepers face, are restricted to the US^10^. Even as the demand for commercial pollination services continues to grow, the ecological influences of modern agriculture on honey bee health, with its intensive cropping practices and extensive monocultures that include single to a few cultivars, are compounding the problematic impacts of these factors on honey bee populations.

Honey bees are generalist pollinators having mutualistic relationships with several plant species for their nutritional requirements that ensures normal growth and development of workers and queens^11,12^. Foraging bees acquire specific nutrients and adapt to changes in the foraging habitat resulting in efficient gathering of pollen and nectar resources from the environment^13–15^. Colonies modulate forager allocation depending on nutritional needs and changes in colony food stores^16–20^. At the individual level, selective foraging depends on the ability of bees to discriminate different nectars and pollens based on multiple sensory stimuli including color, taste, tactile qualities, and odor^15,21–26^. Thus, it is apparent that pollen foragers may be able to select pollen from available resources for nutrient complementarity which can be beneficial for colonies adapting to changing resource landscapes^19,20,25,27–29^.

Almond bloom in California begins in mid-February when there are very few other plants in bloom and foragers from honey bee colonies brought into almond orchards for pollination have limited forage options beyond blooming almonds^30^. It has been shown that honey bees that pollinate monoculture fields may need alternate floral or nutritional supplements to maintain colony health^31^. Incorporating habitats with mixed flowering plant species is an accepted management practice to improve access to diverse floral resources^32^. However, honey bees learn to associate rewarding flowers to specific cues from flowers including odors or colors^33–37^. Studies suggest that when such associations occur, bees are likely to visit flowers with similar colors or odors, increasing foraging efficiency^38^ and pollination efficacy^39^. This further leads to the question of whether bees foraging in a flowering orchard or crop field will visit flowers from nearby flowering habitats. Using approaches based in choice assays to understand forager preferences, we sought to delineate foraging behaviour of bees habituated to pollen from a specific source. Habituation was accomplished by training bees to forage on specific bee-collected pollen from pollinator-dependent crops or from mixed species plantings, allowing us to mimic foraging bees on agricultural lands. Our study thus included bee-collected pollens from hives in (a) almond orchards, *Prunus amygdalus*, (b) sunflower fields, *Helianthus annuus*, and (c) Mixed Species Pollen (MSP) including inter-crop mustard-mix wildflower plantings (*Brassica ruvo*, *Sinapis alba*, *Brassica juncea* Nemfix and *Raphanus sativus*) and other flowering species in the vicinity of the hives. We report the foraging choices of bees habituated to training pollen types. Using metabolomic analyses of pollen, we then detail the nutritional composition of bee-collected pollen from these different agricultural sites in California.

## Materials and methods

### Pollen collection for foraging assay

Pollen from colonies placed for pollination in almond orchards, sunflower fields and from MSP plantings were collected during the spring and summer of 2020. The hives for pollen collection were placed in the respective fields in Yolo County, California. During the peak flowering time, 10–15 hives each with pollen traps were placed in blooming almond orchards, blooming sunflower fields, and near blooming pollinator-friendly plantings. The MSP collection was completed well after completion of almond flowering and well before the beginning of sunflower bloom, minimizing the overlap of collected MSP pollen with these crops^40,41^. Pollen was collected in the pollen trap placed at the base of the bee hive [Shaparew model; US 4,337,541 A^42^]. The activated traps collected pollen from returning foragers up to ten days during the respective blooms. Bee-hive pollen from almond orchards was collected between mid-February–early March 2020, from mixed species plantings in mid-March–early May 2020, and from sunflower fields in July 2020. Bee-collected pollen from the traps were emptied into clean, labeled falcon tubes and stored at − 20 ℃ until further use. Using a kitchen coffee grinder, the pollens from the pollen traps were pulverized^20^, and the grinds strained using a common wire-mesh strainer to obtain ground or crushed pollen. The ground pollen from each of the three fields was divided into subsets of 30 g each, placed in 50 mm × 15 mm, individually weighed and labelled petri dishes used for experimental assays.

### Hive setup

A total of 30 honey bee colonies with approximately 5000 adult workers and a laying queen each, referred to as nucleus colonies, were acquired from a stakeholder apiary in Yolo County, California during April 2021 and they served as the source of experimental colonies and brood frames for the entire period of the study. To represent a normal growing colony, each nucleus colony was given one frame with different stages of developing larvae and pupae, three frames of empty comb, and one frame without a drawn wax comb^43^. The colonies were transferred to individual Quick-Set canopy tents (3.81 m × 3.81 m × 2.38 m pop-up canopy tents; Clam™, Rogers, MN), set up linearly east to west, spaced seven meters apart and secured firmly. The colonies had ad libitum access to water and 27.6% w/v Pro-Sweet syrup (sucrose (50%), fructose (22%), dextrose (27%) and maltose (0.5%) and higher saccharides (0.5%; Mann Lake, Ltd., Woodland, CA), throughout the experimental period. The colonies within the tents did not have access to pollen other than that provided at the feeding stations. Within the tent, the colonies, with their entrances facing south, were set up with pollen feeding stations placed 3.5 m away. Colonies were inspected weekly to assess queen activity, egg-laying, brood rearing, syrup and pollen stores, and for signs of diseases and pests^44^. To maintain colony strength during foraging assays and to ensure continual emergence of adult bees within the tent, all experimental hives were supplemented with one frame of capped (pupal) brood at 21 and 46 days from different source colonies in the apiary. Including frames from different colonies was done to eliminate source colony effect on foraging behavior. During any given training period, there was one colony per pollen type within each tent. For the duration of the experimental period, we had a total of three colonies per pollen type. Choice assays were combined across the three colonies to obtain forager preferences for each training pollen type.

### Training

Immediately after transferring hives into their tents, modifying previously established protocol^15^, bees were provided with the respective training pollen type for one week before beginning choice assays to simulate bees foraging on the specific flowering crop field or orchard thus habituating them to that type of pollen. Training pollen was also provided subsequently between choice assays. The hives were randomly assigned to one of the three training pollen types: H_a_ – hive receiving bee-collected almond pollen, H_s_ – hive receiving bee-collected sunflower pollen and H_m_ – hive receiving bee-collected MSP. Petri dishes with ground pollen were placed at the feeding station in the respective tents and replaced daily with freshly ground pollen. The amounts of pollen collected by foragers per day was calculated by recording the weights of petri dishes with pollen at the beginning and end of each day.

### Pollen choice assays

After the initial week of foraging on training pollen, 3 h choice assays were conducted between 0800 and 1400 h, when the ambient temperatures ranged from 17 to 32 ℃. During the choice assay, the training pollen dish was replaced with three choice pollen dishes, one of each pollen type, arranged in a triangle and spaced 21 cm apart from each other. Each dish with pollen was weighed prior to the assay. Dishes were rotated randomly every 10 min to minimize development of locational preference in foragers^37^. At the end of the 3 h period, the choice assay dishes were replaced with the respective training pollen. The amount of pollen remaining in each choice dish was weighed and the amounts collected by foragers was calculated as a difference between the respective initial and final weights for each petri dish. Choice assays were conducted every other week, with training pollen provided during the weeks when assays were not taking place. A single trial consisted of three choice assays, one assay per hive, done consecutively over three days, giving a total of three assays per pollen type. With a total of seven trials, there were 21 choice assays per pollen type.

### Forager behavior during choice assays

Foragers were observed for pollen sampling behavior using a previously described protocol^37,45^. For trials 1–3, scan sampling was completed by capturing a single photo of all three pollen dishes, every 10 min using an iPhone SE model smartphone camera (Apple Inc. Cupertino, CA). Photos were taken immediately before the rotation of the dishes, with the camera lens perpendicular to the ground at 90 cm from the pollen dishes, and the number of bees at each pollen type was determined from the photos. Bees were assigned to a dish only if they were on the pollen, within the circumference of the dish, clinging to the lip of the dish, or if crawling or flying, at least halfway within the circumference of the dish. For trials 4–7, video recordings were completed for the entire 3-h period using a tripod mounted Canon Inc. (Ōta City, Tōkyō, Japan) EOS 5D Mark IV model camera with the lens perpendicular to the ground at 94 cm from the pollen dishes. The dishes were spaced 16 cm apart in a triangle, to accommodate the field of view of the camera lens and placed atop a 45 cm × 64.5 cm black tray for visual contrast. Scan sampling was done from these videos by capturing screenshots of all three pollen dishes every 10 min and scoring bees as described above.

From the video recordings, the first 35 bee flights were tracked per choice assay. For each flight, the sequence of dish visitations, arrival and departure timestamps, and behavior types exhibited at each visitation were recorded. Bee flights were defined by when the bees flew into and out of the video frame. The following four behavior types were recorded – (i) *arrival*: the moment a flying or hovering bee either arrived around the circumference of the dish or landed on the pollen itself, (ii) *departure*: the moment the same bee initiated fast and directed flight away from the dish. During each visitation – (iii) *foraging*: characterized by pollen packing behavior using their legs and (iv) *assessing*: any behavior besides foraging, including landing atop pollen with no scrabbling, hovering around the dish, or departing soon after arriving. All behavior recordings were completed by the first author, assuring uniformity. The low intensity of foraging activity at the feeders allowed for tracking individual bee movements accurately.

### Statistical analyses of behavioral data

Two-way analysis of variance (ANOVA) with interactions, followed by Tukey’s multiple comparisons were performed using RStudio version 4.1.1.^46^. The independent variables were the training and choice assay pollen types (almond, sunflower and MSP pollens). To determine whether training pollen type affected the relative amounts of choice pollen types collected, the proportion of total pollen collected per type from trials 1–7 was used as the dependent variable. To determine whether bee foraging behavior was affected by training pollen type, the proportion of total bee visits per pollen type from trials 1–7 scan sampling was used as the dependent variable. Scan sampling data from all trials were pooled and grouped by training pollen type. As is the standard procedure to eliminate heteroscedasticity in proportional data^47,48^, proportions of visits were arcsine transformed prior to statistical analysis. Four chi-square tests of independence were performed, using pooled data from trials 4 – 7, to test the randomness of forager flight patterns (See S1 for the R packages used). To assess forager flight patterns, we tested whether proportions of bee arrivals per choice assay pollen dish for each hive was independent of that hive’s training pollen type. The two nominal variables were training pollen type and choice assay pollen types. Pairwise nominal independence tests, without correction were completed to reduce the occurrence of false negatives. Each hive was separately tested for differences between choice assay pollens on the proportions of behaviors observed via video recordings followed by pairwise nominal independence tests with Bonferroni correction. Nominal variables were foraging or assessing behaviors and choice assay pollens.

### Pollen metabolomics

30 bee-collected pollen samples, 10 each from almond (H_a_ hives), sunflower (H_s_ hives) and MSP (H_m_ hives) pollens were profiled using reverse phase and Hydrophilic Interaction Liquid Chromatography (HILIC) coupled with quadrupole time-of-flight mass spectrometry.

### Sample preparation

300 mg of pollen sample was combined with 3 mL of methyl tert‐butyl ether (MTBE):methanol:water (6:3:1, v/v) in an 8 mL vial and vortexed for 60 min at 4 ℃. Three blank samples were also prepared alongside with no pollen but were handled the same as pollen samples throughout extraction. The samples were centrifuged for 0.5 min at 2500 *rpm*, and 500 μL of sample was collected for the ‘Organic phase’ analysis described below. To the remaining sample 625 μL of cold water was added and vortexed for 30 min at 4 ℃ to extract polar metabolites. The samples were centrifuged for 10 min at 2900×*g* to separate aqueous and organic layers. The upper lipid‐rich organic layer was removed, and the lower layer washed twice with MTBE to remove residual lipids from the lower aqueous phase. 3.3 mL of acetonitrile was added to the lower phase to induce protein denaturation and precipitation, the solution was vortexed and centrifuged. 500 μL of the supernatant was collected in a new, clean vial for “Aqueous phase” analysis described below.

### UPLC‐MS analysis

#### Aqueous phase

An aqueous extract of 2 μL was injected onto a waters acquity UPLC system. Separation was achieved using an acquity premier BEH Amide column (1.7 μM, 2.1 × 100 mm), with a gradient from solvent B (95% ACN, 5% Water, 0.1% Formic, 10 mM AmOH) to solvent A (Water, 0.1% Formic, 10 mM AmOH). Injections were made in 10%A, held there for 0.5 min, ramped to 25% A over 6.5 min, ramped to 50% A over 2 min, ramped to 85% A over 1 min, held there for 0.5 min, returned to starting conditions over 1 min and allowed to re‐equilibrate for 3.5 min, with a 500 μL/min constant flow rate. The column and samples were held at 30 ℃ and 6 ℃, respectively. The column eluent was infused into a Waters Xevo G2‐XS Q‐TOF‐MS with an electrospray source in positive ionization mode, scanning 50–1200 m/z at 0.1 s per scan, alternating between MS (6 V collision energy) and MSE mode (15–30 V ramp). Calibration was performed using sodium formate with < 1 ppm mass accuracy. The capillary voltage was held at 700 V, source temperature at 150 ℃, and nitrogen desolvation temperature at 600 ℃ with a flow rate of 1000L/hr.

#### Organic phase

Organic extract of 1 μL was injected onto a Waters Acquity UPLC system. Separation was achieved using a waters acquity UPLC CSH Phenyl Hexyl column (1.7 μM, 2.0 × 100 mm), using a gradient from solvent A (Water, 0.1% ammonium formate) to solvent B (Acetonitrile, 0.1% formic acid). Injections were made in 99% A, held at 99% A for 1 min, ramped to 98% B over 12 min, held at 98% B for 3 min, and then returned to starting conditions over 0.05 min and allowed to re‐equilibrate for 3.95 min, with a 600μL/min constant flow rate. The column and samples were held at 65 ℃ and 6 ℃, respectively. The column eluent was infused into a Waters Xevo G2‐XS Q‐TOF‐MS with an electrospray source in positive mode, scanning 50–1200 m/z at 0.1 s per scan, alternating between MS (6 V collision energy) and MSE mode (15‐30 V ramp). Calibration was performed using sodium formate with 1 ppm mass accuracy. The capillary voltage was held at 700 V, source temperature at 150 ℃, and nitrogen desolvation temperature at 600 ℃ with a flow rate of 1000 L/hr.

#### Pooled quality control (QC) sample

A pooled QC sample was generated for each of the two phases. In each case, a 50 μL aliquot was collected from each independent sample extract, transferred to a single pooled quality control vial, vortex mixed, and aliquoted into six independent autosampler vials. QC injections were made at the beginning of the analytical run, approximately every 7th injection, and at the end of the run. The pooled QC samples were used as a quality control check in data processing, with results described below.

#### Data analysis and statistics for metabolomics analyses

RAMClustR version 1.2.4 in R version 4.1.2^49^ was used to normalize, filter, and group features into spectra.XCMS^50,51^ output data was transferred to a ramclustR object using the rc.get.xcms.data function. Feature data was extracted using the xcms featureValues function. Features which failed to demonstrate signal intensity of at least tenfold greater in QC samples than in blanks were removed from the feature dataset. 2651 of 35,289 features were removed. Features with missing values were replaced with small values simulating noise. For each feature, the minimum detected value was multiplied by 0.1. Noise was then added using a factor of 0.1. The absolute value was used as the filled value to ensure that only non‐negative values carried forward. Features were normalized by linearly regressing run order versus QC feature intensities to account for instrument signal intensity drift. Only features with a regression p‐value less than 0.05 and an r‐squared greater than 0.1 were corrected. Of 32,638 features, 3783 were corrected for run order effects. Features were filtered based on their QC sample Co-efficient of Variation (CV) values. Only features with CV values less than or equal to 1 in MS or MSMSdata sets were retained. 86 of 32,638 features were removed. Features were clustered using the ramclustR algorithm^52^. See S1 for detailed parameter settings and packages used for analyzing behavioral and metabolomic data.

## Results

### Foraging behavior

**Figure 1:**
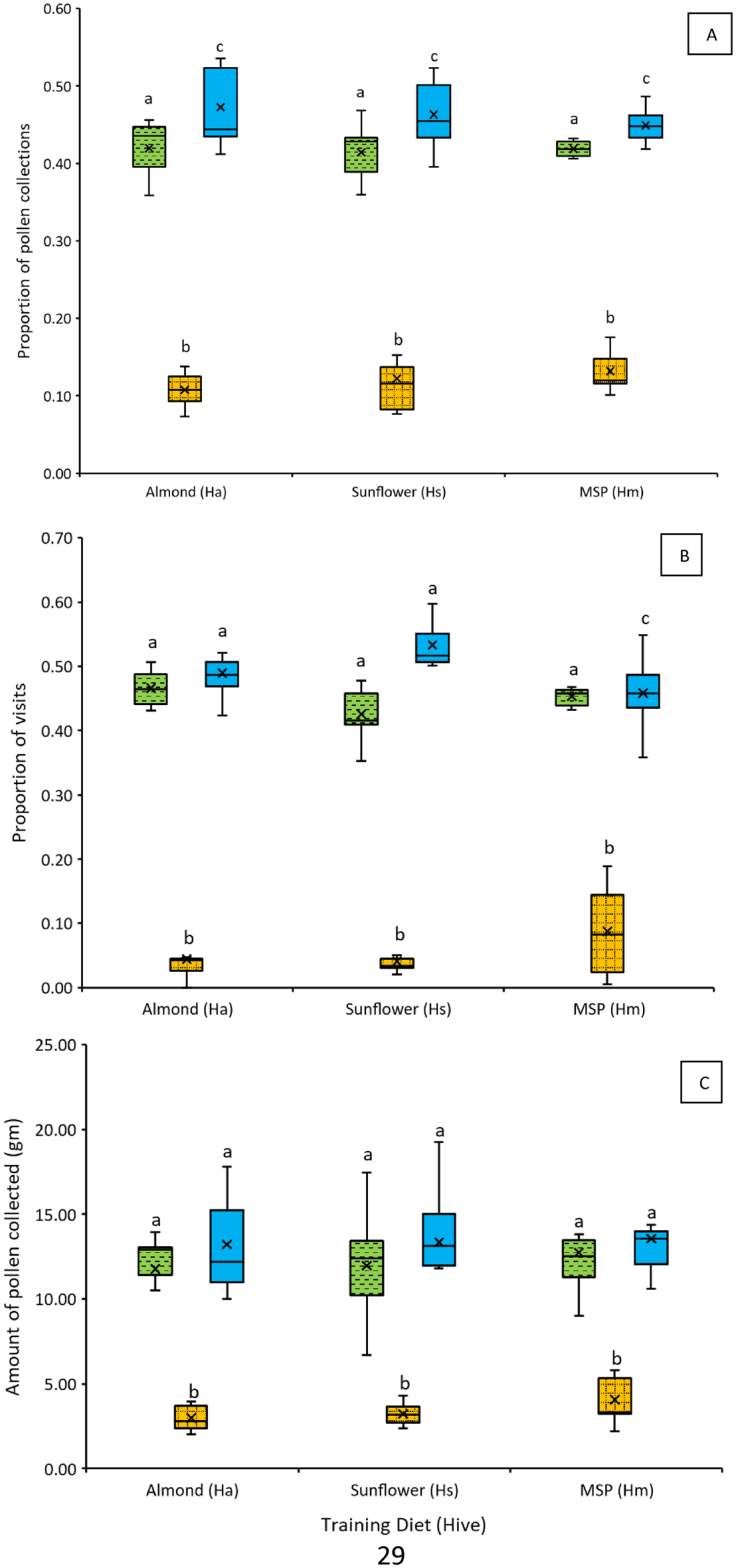
Forager behaviors in the choice arena. (**A**) Proportion of the different pollen types collected, (**B**) proportion of visitation by bees to the different pollen and (**C**) the amount of the different pollen types collected during choice assays. Box plots represent the median (solid line) ± SE and mean (shown as x) of the proportions for the three training diets (hives). Letters indicate significant difference by Tukey’s test.  Almond,  Sunflower,  MSP (mixed species plantings).

We tested the impact of training pollen on foraging choices in honey bees. During the study, the experimental hives (H_a_, H_s_, and H_m_), foraged to similar extents on their training pollen, collecting a total of 195.93 gm, 212.59 gm, and 199.88 gm of training pollen per hive, respectively. Two-way ANOVA indicated that training pollen type significantly affected the amount of pollen collected from different dishes during choice assays (F_(2, 54)_ = 508.56, p < 0.0001; Fig. 1A). Tukey’s post-hoc test of means indicated that regardless of training pollen type, bees collected MSP the most, followed by almond pollen and the least preferred was sunflower pollen (Fig. 1C). The training pollen alone did not significantly affect total pollen collection (F_(2, 54)_ = 0.00, p > 0.9). Furthermore, there was no significant interaction effect of training pollen type with choice assay pollens (F_(4, 54)_ = 0.72, p = 0.58). The experimental hives in the three tents were similarly active during the choice assay as seen by a total of 2976, 3358, and 2773 visits photographed per hive at the respective feeders. Two-way ANOVA demonstrated that choice pollen type significantly affected visitation proportions per dish (F_(54, 2)_ = 553.19, p < 0.0001; Fig. 1B). Tukey’s test showed that bees, irrespective of training pollen type, visited MSP pollen the most, followed by almond and then sunflower pollens. Training pollen type did not significantly affect total visitation (F_(2,54)_ = 0.00, p > 0.9). There was a significant interaction effect between choice assay pollen types and training pollen type (F_(4, 54)_ = 3.994, p < 0.01). For H_a_ and H_m_ hives, the proportion of visits to almond and MSP pollen dishes were not significantly different (Fig. 1B) but for the H_s_ hive, the proportion of bees visiting MSP pollen was greater than those visiting almond pollen, (Fig. 1B). For all training pollen types, sunflower pollen was the least visited and collected (Fig. 1C).

**Figure 2:**
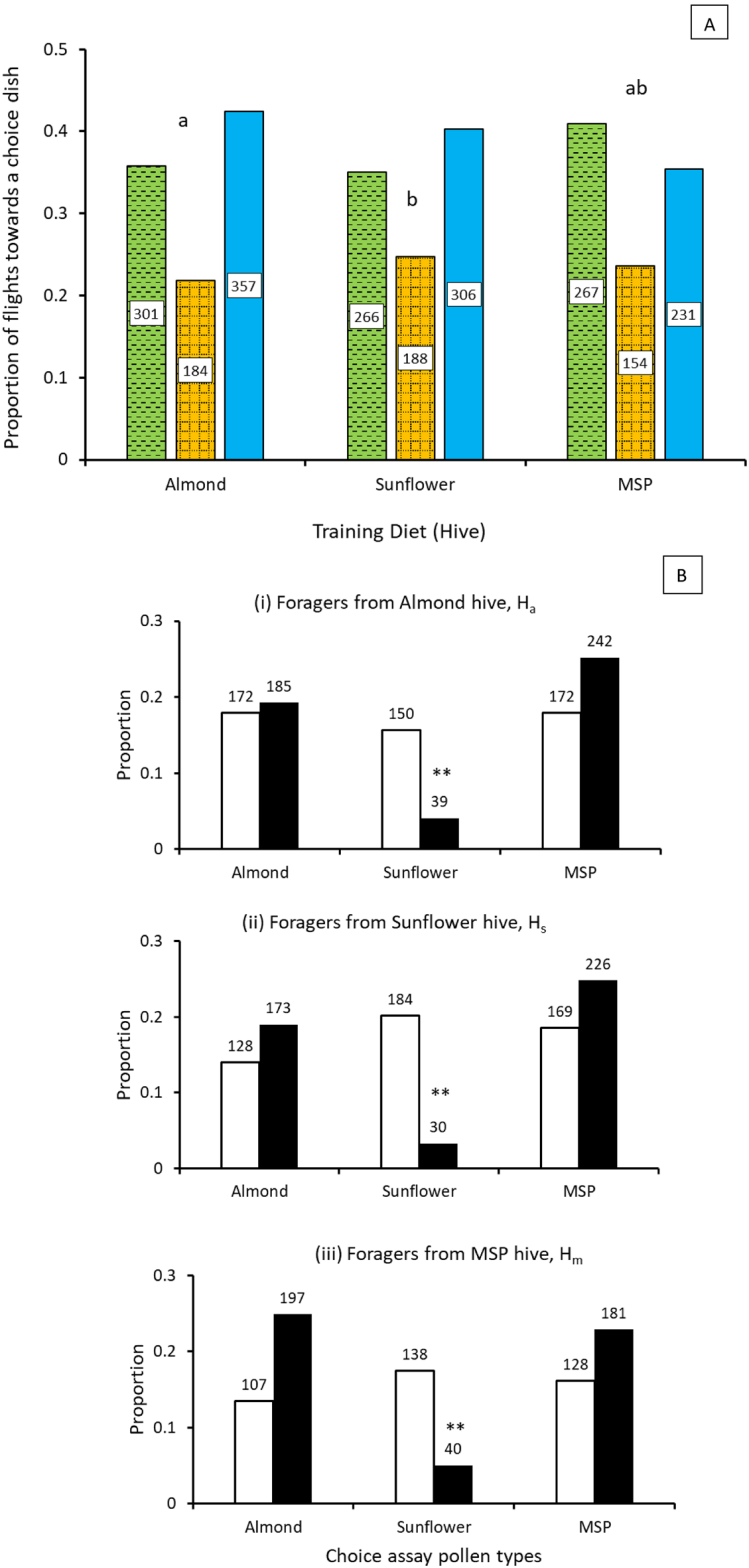
Forager choice patterns (**A**) Bar graphs represent the proportion of flights towards a dish counted as arrivals to that dish. Different letters indicate significant differences (p < 0.04) based on χ^2^ test for observed proportions compared to that expected if arrivals were random. The numbers inside bars represent the number of flights towards the respective dish in the choice array. Almond, Sunflower, MSP (mixed species plantings). (**B**) Bar graphs represent the proportion of foragers assessing (open bars) or collecting (closed bars). The numbers above the bars are the respective sample sizes for assess and collect behaviors at each dish. Observed behaviors were significantly different from expected random (i) χ^2^_(2, 960)_ = 76.75; (ii) χ^2^_(2, 791)_ = 123.21; (iii) χ^2^_(2, 910)_ = 87.41. **Significant at p < 0.00001.

The χ^2^ test of independence was non-significant indicating that the arrival flights were not independent of the training pollen type and preference during choice assays depended on the training pollen type (χ^2^_(4, 2254)_ = 9.99, p = 0.04; Fig. 2A). Pairwise nominal independence testing with Bonferroni correction showed significant differences between hives H_a_ and H_m_ (χ^2^_(4, 2254)_ = 9.99, p = 0.02) suggesting that when given a choice, bees previously trained on almond pollen flew more often towards MSP pollen. Conversely, bees trained on MSP pollen flew more often towards almond pollen (Fig. 2A). Bees from both H_a_ and H_m_ hives approached sunflower pollen least frequently. Bees from H_s_ hive also flew to MSP pollen most often and to sunflower pollen least often (Fig. 2A). However, differences between bees from H_s_ and H_a_ hives (χ^2^_(4, 2254)_ = 9.99, p = 1.0) and H_s_ and H_m_ (χ^2^_(4, 2254)_ = 9.99, p = 0.18) were not significant.

To test whether training pollen type affects behavioral responses of bees to pollen in the choice assays, separate χ^2^ tests of independence comparing the frequency of foraging versus assessment behaviors at each choice pollen type were conducted for each training pollen type and the frequency of behaviors was not different from expected for all hives (p < 0.0001; Fig. 2B). Bonferroni-adjusted pairwise nominal independence tests demonstrated that regardless of training pollen type, visitations more often resulted in foraging behavior at almond and MSP pollen whereas visits to sunflower pollen was less frequent (p < 0.0001). For all training pollen types, there were no significant differences in the frequency of foraging versus assessing behaviors between almond and MSP pollens (p > 0.05).

### Pollen metabolomic analysis

**Figure 3:**
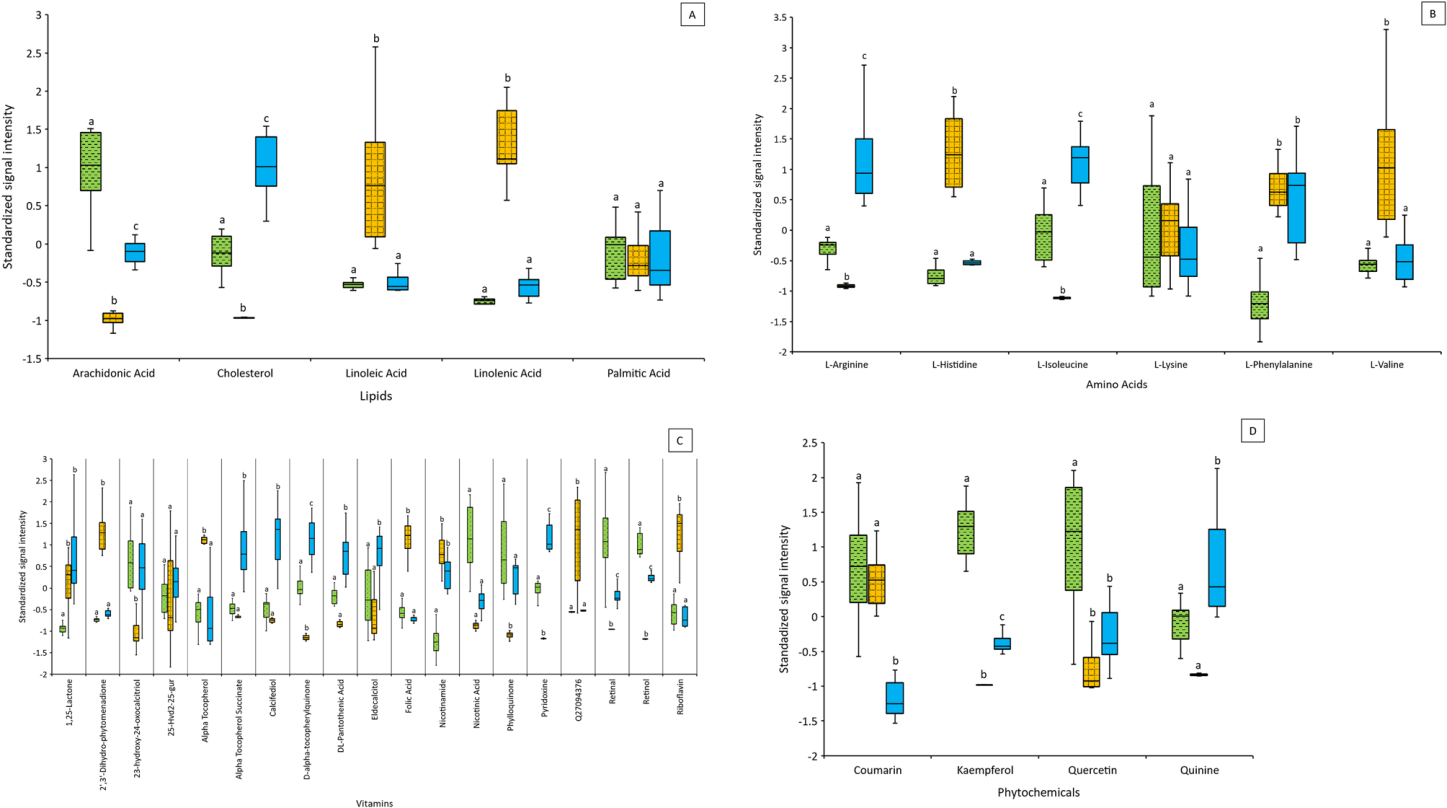
Metabolomic profiles of pollen types used in the choice assays and as training diets. (**A**) Fatty acids profiles; (**B**) a few essential amino acids (EAAs) leucine, methionine, threonine, and tryptophan are EAAs but were not captured by RPLC or HILIC analyses; (**C**) Vitamins and/or their analogs; (**D**) a select few beneficial phytochemicals. Box plots represent the median (solid line) ± SE of standardized signal intensities. Different letters indicate significant differences within individual nutrients across pollen types.  Almond,  Sunflower,  MSP (mixed species plantings).

Pollen is a complex mix of several different chemical compounds, all of which have not been fully annotated. The full list of compounds from our analyses are presented in S2. Our results demonstrate extensive chemical diversity of compounds in all the three types of pollen assayed. We present signal intensities of a few distinct fatty acids (FA; Fig. 3A), essential amino acids (EAA; Fig. 3B), vitamins (Fig. 3C) and phytochemicals (plant secondary compounds; Fig. 3D). Strength of signal intensities is directly related to the concentration of these chemicals in the pollen.

Of the different compounds grouped under lipids and FAs, cholesterol levels were highest in MSP pollen followed by almond pollen with sunflower pollen showing the least. Almond pollen showed high signal intensities for arachidonic acid and sunflower pollen exhibited highest signal intensities for linoleic and linolenic acids (omega 3 and 6 fatty acids, respectively). Signal intensities for palmitic acid were similar for all the three pollens (Fig. 3A).

Of the different EAAs, six essential amino acids, as described by deGroot^53^ were assessed. Leucine, methionine, threonine and tryptophan were not captured by RPLC or HILIC. Signal intensities for arginine and isoleucine were lowest in sunflower pollen and highest in MSP pollen. Signal intensities for histidine, and valine were similar for almond and MSP pollens, and significantly lower than intensities for these EAAs in sunflower pollen. While all the three pollens had similar signal intensities for lysine, almond pollen had lower signal intensity for phenylalanine than sunflower and MSP pollens, which had similar signal intensities (Fig. 3B).

With regards to vitamins, almond and MSP pollens exhibited detectable signal intensities in most of the compounds annotated. Almond pollen exhibited the highest signal intensities for retinol (vA) and nicotinic acid (vB_3_). Sunflower pollen exhibited the highest signal intensities for riboflavin (vB_2_), folic acid (B_9_), alpha-tocopherol (E), and the vitamin K metabolite 2′3’-dihydro-phytomenadione. MSP pollen exhibited the highest signal intensities for pantothenic acid (vB_5_) and pyridoxine (vB_6_), five of six measured vitamin D_3_ metabolites, vitamin E metabolites, succinate and D-alpha-tocopherylquinone. Phylloquinone (vK_1_) signal intensity in sunflower pollen was less than that of almond and MSP pollens, which were similar. Signal intensity for nicotinamide (vB_3_) in almond pollen was less than that of sunflower and MSP pollens, which were similar. Whereas vitamins A and D3 showed the highest signal intensities in almond and MSP pollens, other vitamins were variably distributed in these pollens (Fig. 3C).

Phytochemicals or plant secondary metabolites are a range of different compounds grouped under steroids, flavanols, alkaloids and phenols. We focus our results here on phytochemicals that have been shown to have benefits for honey bees. Our analyses detected similar signal intensities for coumarin in almond and sunflower pollens, with a significantly lowered intensity in MSP pollen. Almond pollen showed the highest signal intensities for kaempferol and quercetin, which were both low in sunflower and MSP pollens. Signal intensities for quinine was the highest in MSP pollen. Quinine in almond pollen was a distant second in signal intensity, and not significantly different from that in sunflower pollen (Fig. 3D).

## Discussion

In the present study, honey bees were trained to forage on bee-collected hive pollen from colonies placed in blooming fields of almond, sunflower and mixed species wildflower habitats, reflecting the diet available for pollinators in the seasonal agricultural landscapes of central California. Our results show that bees preferred the pollen collected from the mixed species plantings over that from sunflower fields and almond orchards, and they continued to collect all three when given a choice. It is to be noted that all the pollen used for training and choice assays were blends with the expectation that hive-collected pollen from almond orchards is likely to be predominantly almond pollen, and that from sunflower fields is predominantly sunflower pollen. Metabolomic analyses of the bee-collected pollen from the different sites document the nutrient composition of the specific hive-collected pollens. A closer look at the behavioral preference or lack thereof, shows that there are multiple steps involved including bees transitioning between feeding stations, actively assessing pollen types before collection, and returning to a dish that was previously passed over after having collected from another pollen type. Such re-visitation of a rejected dish is indicative of the process by which generalist bees incorporate plant species diversity into their foraging route and track available resources in a foraging arena^38,54^. Polylecty, generalized floral preference by pollinators, allows for close surveillance and exploitation of diverse floral resources, maintaining constancy to a rewarding patch or species while tracking the ongoing phenological fluxes^55^. Although forager behavior in our study could not be expanded to the palynological diversity in what bees brought back to their hives, it is still interesting that we did not observe clear choice-dish constancy^38,56^ even with as few as three choices, supporting suggestions that floral constancy in bees involves more complex processes than just the number of choices^57,58^.

While it may be argued that sunflower, a late summer (July–August) crop in California is a phenological mismatch with almond and MSP, bees in our choice assays collected pollen from all the three dishes albeit in different amounts, suggesting that being generalist foragers^55^, honey bees gather any available pollen and a diversity of pollen sources may likely be preferred. Almonds bloom from mid-February through mid-March, and the mixed species inter-crop plantings bloom between March through June. Our results showing preference to MSP may hypothesize nutritive complementarity that bees may derive from species mixes^13,15^ but our study was not explicitly designed to test this.

Our study, unique in that we explore the metabolomic profiles of the hive-collected pollen, provides detailed information on the nutritive compositions of the pollen within hives. The two most preferred pollen types in our study were low in linolenic and linoleic acids, the two essential polyunsaturated fatty acids (ePUFAs). The signals reported represent free linoleic and linolenic acids, and the data do not explicitly indicate the relative abundances of these two compounds within the lipid-fatty acid composition. The signal intensity values of these ePUFAs for almond and MSP pollens were about 4 to16 times lower than that for sunflower pollen respectively. Bees fed diet poor in essential omega-3 PUFAs experienced detrimental effects on learning abilities and smaller hypopharyngeal glands^59^. Despite the essential nature of these ePUFAs for honey bee reproduction, physiological development, cognition and immune function^60^, lack of preference for sunflower pollen suggests that bees may not be able to detect these compounds and modulate their foraging accordingly. It is also equally likely that the foragers may prioritize other compounds such as total proteins that were not analyzed in our study. Previous studies have termed sunflower pollen as “low quality” as the pollen is relatively low in its protein content in comparison with other important bee forage plants^61,62^. Furthermore, in support of “low quality”, pollen from sunflowers may also have lower than the minimum required amounts of two essential amino acids, methionine and tryptophan, for honey bees^62^. Linolenic and linoleic acids constitute less than 8% of the total lipid content of 6-day old larvae and adult worker bodies^60^. Honey bee pollen foragers learn subtle variations in pollen fatty acid concentration, via olfaction and gustation, and adjust foraging efforts to balance their intake^13,25,29,37^. Tying this together with the behavioral preferences exhibited by foragers, there likely may be a nutritional basis to foraging preferences, but the mechanisms relating to the perception of nutrients is still not well understood.

In contrast, preferred pollen types were characterized by higher levels of free arachidonic acid, an important fatty acid that contributes to innate immunity and worker body weight but is likely detrimental to when present in pollen above optimal thresholds (4% of total lipids)^63^ but unfortunately, our data do not explicitly detect its relative abundance. While it seems that forager preferences may be related to levels of ePUFAs in pollen, these compounds are not present in isolation. A combination of factors including other nutrients and chemicals in the environment could therefore impact detection and preference. We cautiously suggest that, with regards to fatty acids specifically, the observed preferences to MSP and almond pollens may relate to the ability of honey bees to balance appropriate levels of ePUFAs to meet nutritional requirements while minimizing physiological costs. The relative cholesterol of preferred pollen, however, may be of importance as the ecdysteroid, a precursor necessary for adult survival, brood development, and ovary maturation, is not synthesized de novo in insects and must be sourced dietarily^11,64,65^. It is unlikely that honey bees perceive concentration differences as measured via antennation^25^ but given their ability to respond to sugars, amino acids, salts and other bitterants through their chemoreceptors^22,66^, further research on sterol perception could shed light on the impact of pollen cholesterol content on real-time foraging decisions.

Preference for essential amino acids (EAAs) was not consistent across pollen types and bees may be exhibiting nutrient-specific balancing for this class of nutrients crucial for adult bee growth, brood rearing, and thoracic muscle development^18,53^. Earlier studies have demonstrated that honey bees perceive concentration differences in amino acid and fatty acids and learn to forage from complementary sources of amino acids but these relied on bees deprived of specific amino acids prior to testing for preferences on artificial diets^15^, and their ability to discriminate was demonstrated via olfaction mediated proboscis extension responses^25^. The benefits of receiving pollen that was supplemented with the 10 essential amino acids in the Hoover, et al.^67^ study did not reveal clear differences as these colonies had access to natural pollen. Previous studies also showed that the lack of amino acids such as arginine, histidine, lysine, phenylalanine, and valine in the diet for 7 to 14 days resulted in reduced growth in newly emerged bees^53^. Accordingly, colonies predominantly exposed to sunflowers, potentially those colonies pollinating sunflower fields, experience deficiency in arginine but obtain a good share of histidine. Almond pollen was low in valine and colonies receiving only pollen from blooming almond orchards thus may experience compromised growth of newly emerged bees. By utilizing bee-collected hive pollen, foragers in our study experienced a complex mixture of compounds in pollen, rendering minute differences likely undetectable but prolonged exposure to these monocrop pollen, during pollination services, could result in negative impacts on colony development. Vitamins are relatively less understood micronutrients in insects, specifically honey bees but vitamins A and K known to support improved brood production, may not be essential for honey bees^68^.

Phytochemicals, have lately received attention given their evolutionary significance in plant-pollinator mutualisms^69,70^ and we are now beginning to understand the diverse profiles of phytochemicals in plant pollen^71,72^. High levels of quercetin and kaempferol, have been shown to impart health benefits for bees ranging from tolerance to pathogen and pesticide, to improved gut microbiome abundance and adult longevity^73–77^. On the other hand, high levels of coumarin and the quinine-like-compounds seen in our annotation, have been associated with toxicity and aversion exerting a phagoinhibitory effect through attenuation of positive rewards and acquired memory of diets associated with post-ingestive malaise^78,79^. The observed persistence in preference for coumarin and quinine-like-compound rich pollen types, imply that either the concentrations of these compounds in pollen is low enough as to not induce toxicity or that the repulsive qualities may be overshadowed by synergies with other constituents of pollen.

Our study is distinctive in its detailed information on the nutritional content of pollen from hives pollinating almonds, a major pollination-dependent crop, that grosses the highest revenue and sunflowers, the 2nd biggest revenue source for honey bee pollination services in the US^80^. Honey bees are present globally and the intensive agriculture in the US, specifically in the regions of the central valley in California where almond production reached global maxima, present some of the significant challenges to honey bee colonies. Annually in the US, over a million hives are needed in this region to pollinate more than a million acres (4000 km^2^) of flowering almonds^81^. Our results complement the growing literature that demonstrate the benefits of diverse floral resources through inter-row wildflower habitat plantings in intensive agricultural landscapes and draw attention to the pollen nutritional complementarity with the pollination-dependent crops and mixed species of flowering plants.

### Supplementary Information

Supplementary Information 1.

Supplementary Information 2.

## Data Availability

All data generated or analysed during this study are included in this published article [and its Supplementary Information files]. The datasets used and/or analysed during the current study are also available from the corresponding author on reasonable request.
