# Author Correction: Pollen foraging preferences in honey bees and the nutrient profiles of the pollen

**DOI:** 10.1038/s41598-024-68139-7

**Published:** 2024-07-30

**Authors:** Seiji C. Yokota, Corey Broeckling, Arathi H.S. (Seshadri)

**Affiliations:** 1 Invasive Species and Pollinator Health Research Unit, USDA-ARS/PWA/WRRC, Davis, CA 95616 USA; 2 Analytical Resources Core/Data Science Research Institute/Department of Agricultural Biology, Colorado State University, Fort Collins, CO 80523 USA; 3 Pollinator Health in Southern Crops Ecosystems Research Unit, USDA-ARS/SEA, Stoneville, MS 38776 USA

Correction to: *Scientific Reports* 10.1038/s41598-024-65569-1, published online 01 July 2024

The original version of this Article contained an error in Figure 2a where bars for ‘Almond’, ‘Sunflower’ and ‘MSP’ were swapped. The original Figure 2a and accompanying legend appear below.

**Figure 2:**
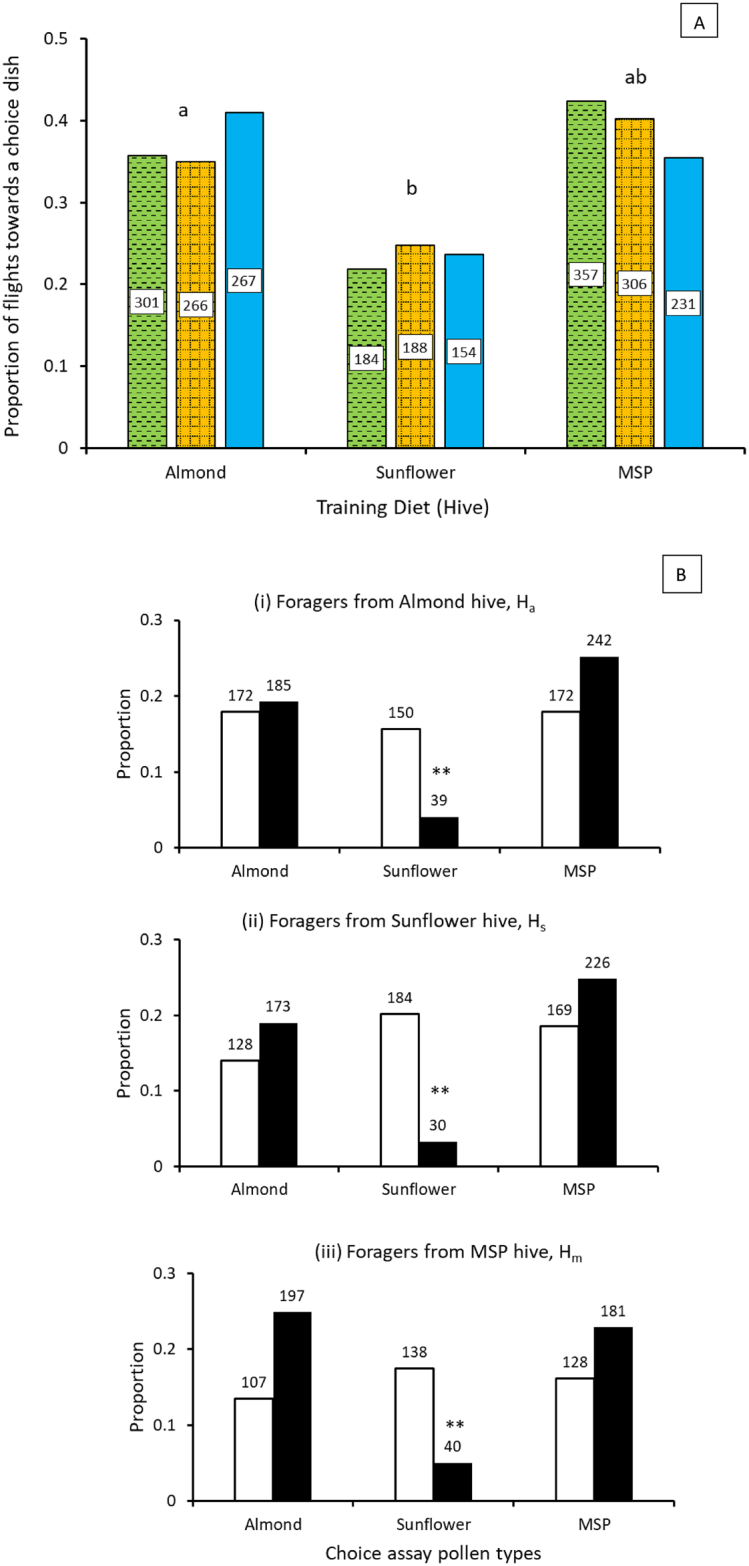
Forager choice patterns (**A**) Bar graphs represent the proportion of flights towards a dish counted as arrivals to that dish. Different letters indicate significant differences (p < 0.04) based on χ^2^ test for observed proportions compared to that expected if arrivals were random. The numbers inside bars represent the number of flights towards the respective dish in the choice array. Almond, Sunflower, MSP (mixed species plantings). (**B**) Bar graphs represent the proportion of foragers assessing (open bars) or collecting (closed bars). The numbers above the bars are the respective sample sizes for assess and collect behaviors at each dish. Observed behaviors were significantly different from expected random (i) χ^2^_(2, 960)_ = 76.75; (ii) χ^2^_(2, 791)_ = 123.21; (iii) χ^2^_(2, 910)_ = 87.41. **Significant at p < 0.00001.

The original Article has been corrected.

