# Supplementary material for: Pollen foraging preferences in honey bees and the nutrient profiles of the pollen
Source: Sci Rep. 2024 Jul 1;14:15028. doi: 10.1038/s41598-024-65569-1 (PMC11217361; doi:10.1038/s41598-024-65569-1)
Supplement: Supplementary file 1 — Supplementary Information 1. [file 41598_2024_65569_MOESM1_ESM.docx]

**R packages used**

**Behavioral data:**

dplyr v1.0.7 (Wickham et al. 2021); ggplot2 (Wickham 2016) packages; rcompanion v2.4.6 (Mangiafico 2021); dplyr v1.0.7 (Wickham et al. 2021); ggplot2 (Wickham 2016); grid and stats (Team 2021); pwr 1.3-0 (Champely 2020); DescTools 0.99.44 (Signorell. 2021); RVAideMemoire packages 0.9-81-2 (Hervé 2021)

**Metabolomics analyses:**

Parameter settings were as follows: st = 2.09, sr = 0.7, maxt = 209, deepSplit = FALSE, hmax = 0.3, minModuleSize = 2, and cor.method = pearson. Molecular weight was inferred from in‐source spectra (Broeckling et al. 2016) using the do.findmain function, which calls the interpretMSSpectrum package (Jaeger et al. 2016). Parameters for do.findmain were set to: mode = positive, mzabs.error = 0.005, ppm.error = 10, ads = default, scoring = auto, and use.z = TRUE. MSFinder (Tsugawa et al. 2016) was used for spectral matching, formula inference, and tentative structure assignment, and results were imported into the RAMClustR object. Annotations were assigned using the RAMClustR annotate function. Annotation priority was assigned from highest priority to lowest: MSFinder spectrum search, MSFinder structure, MSFinder formula, interpretMSSpectrum M. Compounds were assigned to chemical ontogenies using the ClassyFire API (Djoumbou Feunang et al. 2016).

Broeckling, C. D., A. Ganna, M. Layer, K. Brown, B. Sutton, E. Ingelsson, G. Peers, and J. E. Prenni. 2016. Enabling Efficient and Confident Annotation of LC−MS Metabolomics Data through MS1 Spectrum and Time Prediction. Analytical Chemistry **88**:9226-9234.

Champely, S. 2020. pwr: Basic Functions for Power Analysis. R package version 1.3-0. .

Djoumbou Feunang, Y., R. Eisner, C. Knox, L. Chepelev, J. Hastings, G. Owen, E. Fahy, C. Steinbeck, S. Subramanian, and E. Bolton. 2016. ClassyFire: automated chemical classification with a comprehensive, computable taxonomy. Journal of cheminformatics **8**:1-20.

Hervé, M. 2021. RVAideMemoire: Testing and Plotting Procedures for Biostatistics. R package version 0.9-80.

Jaeger, C., F. Hoffmann, C. A. Schmitt, and J. Lisec. 2016. Automated Annotation and Evaluation of In-Source Mass Spectra in GC/Atmospheric Pressure Chemical Ionization-MS-Based Metabolomics. Analytical Chemistry **88**:9386-9390.

Mangiafico, S. 2021. rcompanion: Functions to Support Extension Education Program Evaluation. R package version 2.4.6.

Signorell., A. 2021. DescTools: Tools for descriptive statistics. R package version 0.99.44.

Team, R. C. 2021. R: A language and environment for statistical computing. . R Foundation for Statistical Computing, Vienna, Austria.

Tsugawa, H., T. Kind, R. Nakabayashi, D. Yukihira, W. Tanaka, T. Cajka, K. Saito, O. Fiehn, and M. Arita. 2016. Hydrogen Rearrangement Rules: Computational MS/MS Fragmentation and Structure Elucidation Using MS-FINDER Software. Analytical Chemistry **88**:7946-7958.

Wickham, H. 2016. . ggplot2: Elegant Graphics for Data Analysis. . Springer-Verlag New York.

Wickham, H., R. François, L. Henry, and K. Müller. 2021. dplyr: A Grammar of Data Manipulation. R package version 1.0.7.
